# Synthesis of Wurtzite Cu_2_ZnSnS_4_ Nanosheets with Exposed High-Energy (002) Facets for Fabrication of Efficient Pt-Free Solar Cell Counter Electrodes

**DOI:** 10.1038/s41598-017-18631-0

**Published:** 2018-01-10

**Authors:** Xiaoyan Zhang, You Xu, Junjie Zhang, Shuai Dong, Liming Shen, Arunava Gupta, Ningzhong Bao

**Affiliations:** 1 State Key Laboratory of Material-Oriented Chemical Engineering, College of Chemical Engineering, Nanjing Tech University, Nanjing, Jiangsu 210009 P. R. China; 2 School of Physics, Southeast University, Nanjing, Jiangsu 211189 P. R. China; 3 Center for Materials for Information Technology, The University of Alabama, Tuscaloosa, Alabama 35487 USA; 4 Jiangnan Graphene Research Institute, Changzhou, Jiangsu 213159 P. R. China

## Abstract

Two-dimensional (2D) semiconducting nanomaterials have generated much interest both because of fundamental scientific interest and technological applications arising from the unique properties in two dimensions. However, the colloidal synthesis of 2D quaternary chalcogenide nanomaterials remains a great challenge owing to the lack of intrinsic driving force for its anisotropic growth. 2D wurtzite Cu_2_ZnSnS_4_ nanosheets (CZTS-NS) with high-energy (002) facets have been obtained for the first time via a simple one-pot thermal decomposition method. The CZTS-NS exhibits superior photoelectrochemical activity as compared to zero-dimensional CZTS nanospheres and comparable performance to Pt counter electrode for dye sensitized solar cells. The improved catalytic activity can be attributed to additional reactive catalytic sites and higher catalytic reactivity in high-energy (002) facets of 2D CZTS-NS. This is in accordance with the density functional theory (DFT) calculations, which indicates that the (002) facets of wurtzite CZTS-NS possess higher surface energy and exhibits remarkable reducibility for I_3_^−^ ions. The developed synthetic method and findings will be helpful for the design and synthesis of 2D semiconducting nanomaterials, especially eco-friendly copper chalcogenide nanocrystals for energy harvesting and photoelectric applications.

## Introduction

With recent great progress of both traditional (silicon, thin film solar cells, *etc*.) and emerging 3^rd^ generation solar cells (dye-sensitized, quantum dot, and perovskite solar cells, *etc*.), photovoltaics now provide one of lowest-cost options for future electricity generation^1,2^. In order to obtain solar cells with lower price and good stability, the replacement of noble metal electrode materials has been a long term of concern^3–7^. Noble metal platinum (Pt) has been widely used as counter electrode (CE) materials for 3^rd^ generation solar cells (e.g. dye-sensitized solar cells (DSSCs)). However, the high cost and low natural abundance hinder its large scale industrial applications. To address this issue, many alternative Pt-free materials have been proposed to be used as CE catalysts for DSSCs, including carbon materials, conductive polymers, and inorganic compounds, *etc.* ^6–9^. Potential high efficient CE materials should provide both high electrocatalytic activity and electrical conductivity, as the CE behaves both as a photocatalyst and an electron collector^6^. It is well known that the catalytic and electrical properties of CE materials depend critically on its morphological and structural characteristics such as shape, orientation, and crystal symmetry, *etc.* ^10–12^. Thus, the controlled synthesis of semiconductor materials with proper structure-related catalytic and electrical properties are essential for high efficient Pt-free CE materials.

Recently, two-dimensional (2D) materials (nanosheets/plates) have been the subject of intensive study since the discovery of graphene^13,14^. The large lateral size and atomic thickness of 2D nanomaterials endow them with ultrahigh specific surface area, abundant edge sites, and high percentages of the surface atoms^14–17^, which makes them very attractive for catalytic applications^10,11,15,18–22^. As one of the important p-type semiconductors, Cu_2_ZnSnS_4_ (CZTS) has been proved to be a suitable Pt-free CE material due to the earth-abundant composition, low-toxicity, and high catalytic activity, *etc.* ^23,24^. Although CZTS nanocrystals have been synthesized in shapes of zero dimensional quantum dots and one dimensional arrows, ellipsoids, rods, bullets, rice, *etc*.^25–28^, the formation of more attractive 2D geometries remains challenging. According to crystal growth theory, high energy crystal facets tend to disappear rapidly during the crystal growth process, as the growth rate perpendicular to a high-energy facet is much faster than that along the normal direction of a low-energy facet^29–31^. Various methods, such as solvothermal, pulsed laser deposition, and spray pyrolysis have been used to form CZTS nanosheets/nanoplate arrays on substrates^21,22,32–34^, while the complicated synthesis procedure and the vacuum based process hinder their wide applications. The wet-chemical syntheses have been considered as a class of convenient and reproducible strategies for the preparation of 2D nanomaterials in high yield and large amount^13,35^. Zhang *et al*. have prepared ~14 nm thick chalcogenide nanoplates with composition of CuInS_2_, CuIn_x_Ga_1-x_S_2_, and Cu_2_ZnSnS_4_ via the cation exchange reaction using pre-synthesized CuS nanoplates as template^36^. Up to now, the direct synthesis of ultrathin 2D CZTS nanosheets using wet chemical method remains a challenging task.

Except for the morphology of nanocrystals, the crystal symmetry and orientation are known to greatly influence the physical and electrical properties of materials^37–40^. CZTS usually crystallizes in the kesterite or stannite phase, which have a tetragonal crystal cell^24–26,28^. The metastable wurtzite phase has gained great attention due to the flexibility of stoichiometry and the ability to tune the Fermi energy over a wide range^37^. Phase-transition-driven grain growth of metastable wurtzite CZTS nanocrystals enables its application in the photovoltaic field^38^. Hall-effect measurements of wurtzite CZTS films indicate a higher carrier concentration and a lower resistivity as compared to kesterite CZTS, which means a higher electrical conductivity^12,39^. Theoretical calculations also indicate the delocalized character of Cu *d* states of CuInSe_2_ due to wurtzite-type symmetry, which enhances the abilities of both electron excitation and transport^40^. Thus, the wurtzite phase of CZTS is more attractive for catalytic applications. However, the colloidal synthesis of 2D CZTS nanosheets with wurtzite structure is yet to be realized.

Based on our previous work, we have built up the method to synthesize a series of ternary and quaternary chalcogenide nanocrystals with controlled crystal phase (wurtzite, zinc blend, and kesterite)^28,41,42^, and morphology (rods, rice-like, bullets, *etc*.). The facet- and shape- dependent electrical properties have been investigated for binary PbS nanocrystals^43^. Herein, we report on the colloidal synthesis of wurtzite CZTS nanosheets (CZTS-NS) with exposed high-energy (002) facets via a scalable one-pot thermal decomposition method. Wurtzite CZTS-NS with large lateral dimension of 350 ± 50 nm and thickness of ~5 nm demonstrate promising photoelectrocatalytic activity for triiodide ion reduction at a rate comparable to platinum for DSSCs. This is likely due to the reduced charge transfer resistivity (*R*_ct_) and comparable series resistivity for CZTS-NS and Pt. Theoretical calculation indicates that the (002) facets of CZTS-NS have remarkable reducibility for I_3_^−^ ions, which could be explained by the electron clouds interaction between I_1_ atom and (002) surface. The efficient and scalable method for preparation of 2D nanomaterials also paves a new way for synthsis other copper-based chalcogenide nanomaterials, which shows potential for energy harvesting and photoelectric applications.

## Results

### Characterization of CZTS nanosheets

**Figure 1:**
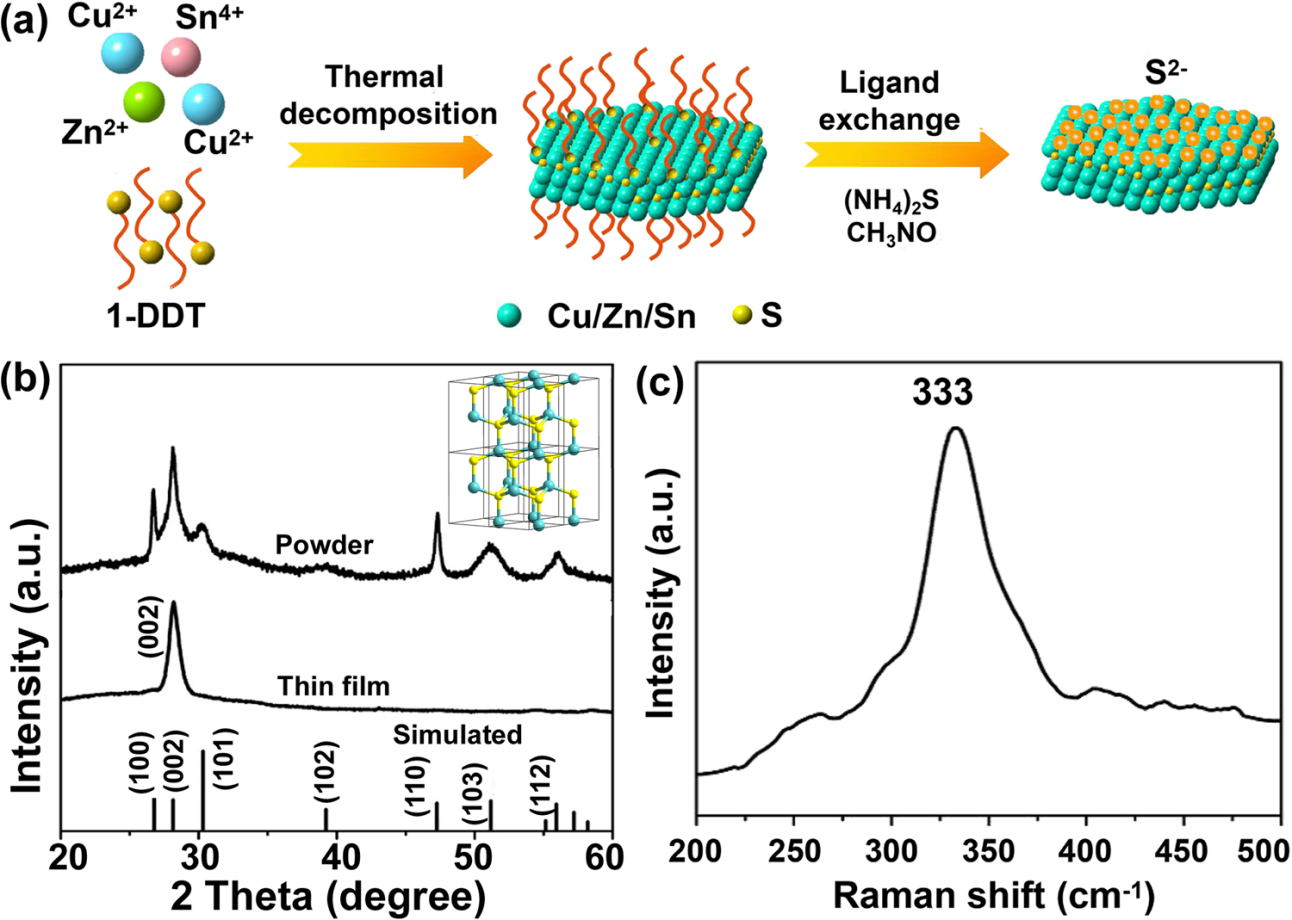
(**a**) Illustration of the formation of CZTS-NS and ligand exchange process, (**b**) XRD patterns of bulk CZTS-NS, stacked thin film, and simulated wurtzite CZTS nanocrystals (inset shows the crystal structure of wurtzite CZTS), and (**c**) Raman scattering pattern of obtained CZTS-NS.

The successful synthesis of wurtzite 2D CZTS-NS takes advantage of using excess 1-DDT as both the sulfur source and reaction solvent. 1-DDT has been widely used as a sulfur source that can balance the reactivity of cations in the solution and passivate the obtained wurtzite nanocrystals^28,41,42,44^. Besides, the reaction solvents can bind to the specific crystal facets and thus force to form nanocrystals with different shapes and orientations. Excess 1-DDT specifically passivating the (002) facets of CZTS nanocrystals can lead to the formation of ultrathin nanosheets. Furthermore, the intermediate products formed with 1-DDT and cations in the reaction system may also contribute to the preferred 2D growth of nanocrystals^45^. Figure 1a shows the illustration of the formation of CZTS-NS and ligand exchange process. The X-ray diffraction (XRD) patterns of CZTS-NS and spray-painted thin films are shown in Fig. 1b. Since no standard XRD pattern for wurtzite CZTS exists in the database, a simulated pattern has been obtained based on the structure (inset of Fig. 1b)^28^. As seen in Fig. 1b, all the diffraction peaks of bulk CZTS-NS match well with the simulated wurtzite CZTS structure (space group *P*6_3_ *mc*; *a* = *b *= 3.843 Å and *c* = 6.337 Å), confirming the wurtzite structure of CZTS-NS. We have noticed that the relative intensity of (100) and (110) peaks are higher than the simulated pattern, which indicates the preferred orientation of CZTS-NS along with the (100) plane. Interestingly, the spray-painted CZTS thin film only exhibits a single characteristic peak at 2*θ* = 28.18°, which demonstrates that the CZTS nanosheet film has a preferred orientation and the (002) facets are preferentially exposed. Due to the similarity of the diffraction peaks for wurtzite CZTS with wurtzite ZnS and monoclinic Cu_2_SnS_3_, Raman spectroscopy studies (Fig. 1c) has been further performed to confirm the phase purity in CZTS-NS. The characteristic Raman peak at 333 cm^−1^ is observed, which is close to the value for bulk CZTS^46^, confirming the single crystalline nature of wurtzite CZTS in the product. No additional peak for other phases of ZnS, CuS, SnS, and Cu_2_SnS_3_ are observed.

In order to improve the conductivity of the as-synthesized CZTS-NS, the bonding molecular species of nanocrystal surface are modified via ligand exchange^47,48^. The FTIR spectra (see Supplementary Fig. S1) of CZTS-NS before and after ligand exchange show that the peaks at 2852 and 2925 cm^−1^ corresponding to C-H stretching in organic ligand almost disappear after ligand exchange, which indicates that the organic ligands on the surface of CZTS-NS have been exchanged with S^2−^. As shown in the inset of Supplementary Fig. S1, the CZTS-NS are transformed from being hydrophobic to hydrophilic, as identified by the color change of hexane (black to colorless) and FA (yellow to black) phase.

**Figure 2:**
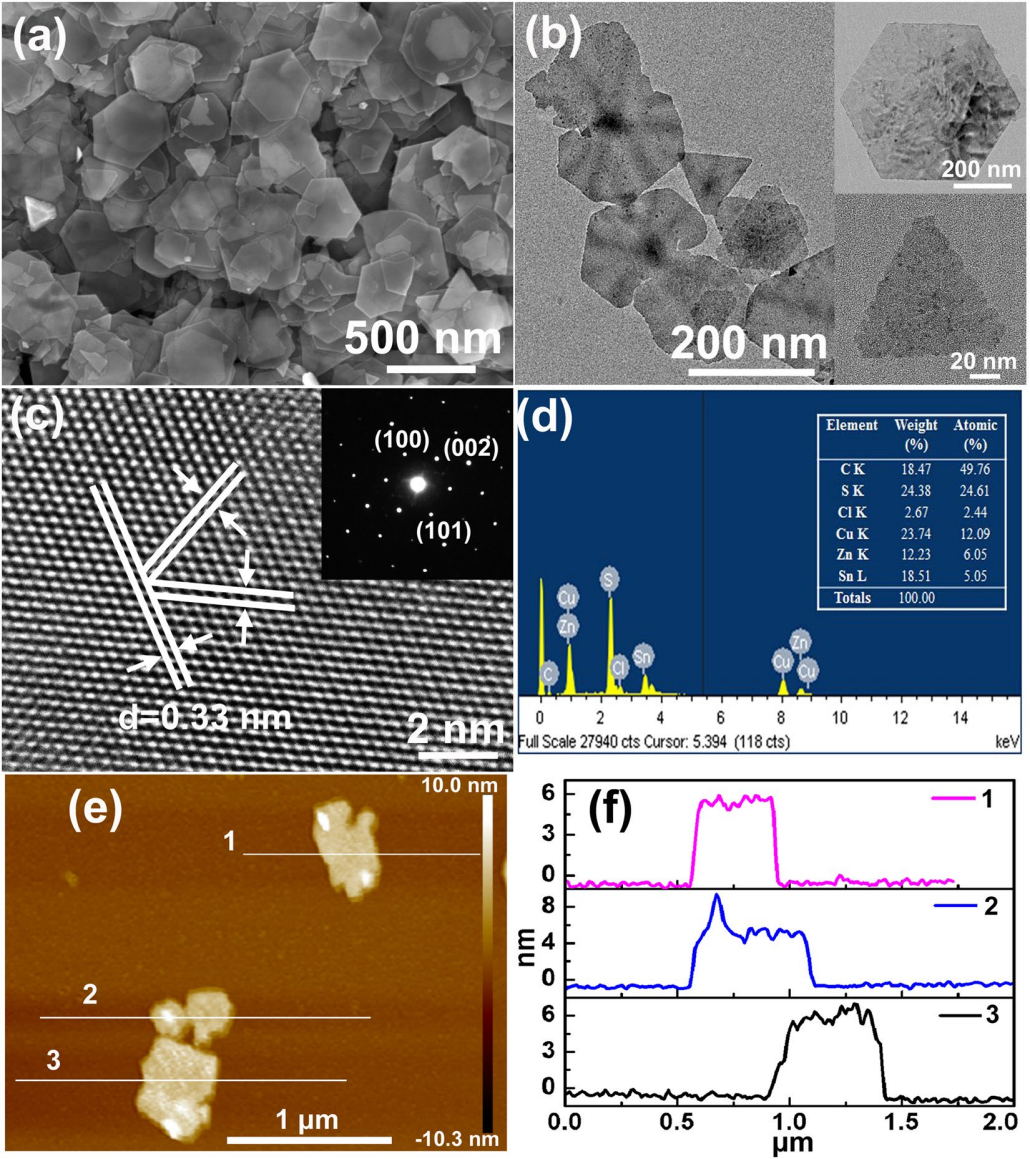
(**a**) SEM image, (**b**) TEM image, (**c**) HRTEM image, (**d**) EDX spectra, (**e**) AFM image, and (**f**) height profiles along the white lines shown in AFM image of CZTS-NS synthesized at 250 ^o^C for 25 min.

The morphology of the as-synthesized CZTS-NS was investigated by using a field emission scanning electron microscope (FESEM) and transmission electron microscope (TEM). As shown in Fig. 2a, the resultant nanosheets appear mainly in hexagonal and quasi-triangular shapes with an average lateral dimension of 350 ± 50 nm, and there also exists some small triangular-shaped nanoplates. The as-obtained CZTS-NS are easily stacked onto each other forming aggregates due to the large surface area. The TEM image of a single CZTS-NS (Fig. 2b) appears bright in color, which indicates that the as-obtained nanosheets are very thin. There are also a small amount of intermediate products such as hexagonal and quasi-triangular shaped nanosheets with irregular ends, similar to the reported growth of djurleite (Cu_1.96_S) nanosheets^45^. The interplanar spacing (*d*-spacing) measured in the high-resolution TEM (HRTEM) image (Fig. 2c) is 0.33 nm, which corresponds to {100} family planes. The angle between (100) and (110) is 60°, corresponding to the hexagonal structure of CZTS. Selected area election diffraction (SAED) pattern of CZTS nanosheets (inset of Fig. 2c) can be indexed to the [001] zone axis of hexagonal CZTS, which indicates the well-crystalized single-crystalline nature of the CZTS-NS. Energy dispersive X-ray spectroscopy (EDX) data of wurtzite CZTS-NS (Fig. 2d) reveals that the average ratio of Cu:Zn:Sn:S is approximately 2.00:1:0.83:4.06, which matches well with the expected elemental ratio of CZTS. Atomic force microscope (AFM) has further been used to measure the thickness of CZTS-NS (Fig. 2e). The height profiles of three selected CZTS nanosheets (Fig. 2f) suggest that the average thickness of the obtained CZTS-NS is about 5.0 ± 0.5 nm, corresponding to 8–9 stacking layers as the height of a unit cell for wurtzite CZTS is about 6.337 Å. To the best of our knowledge, this is the first report of preparation of ultra-thin 2D wurtzite CZTS-NS using one-pot non-injection colloidal chemistry method.

**Figure 3:**
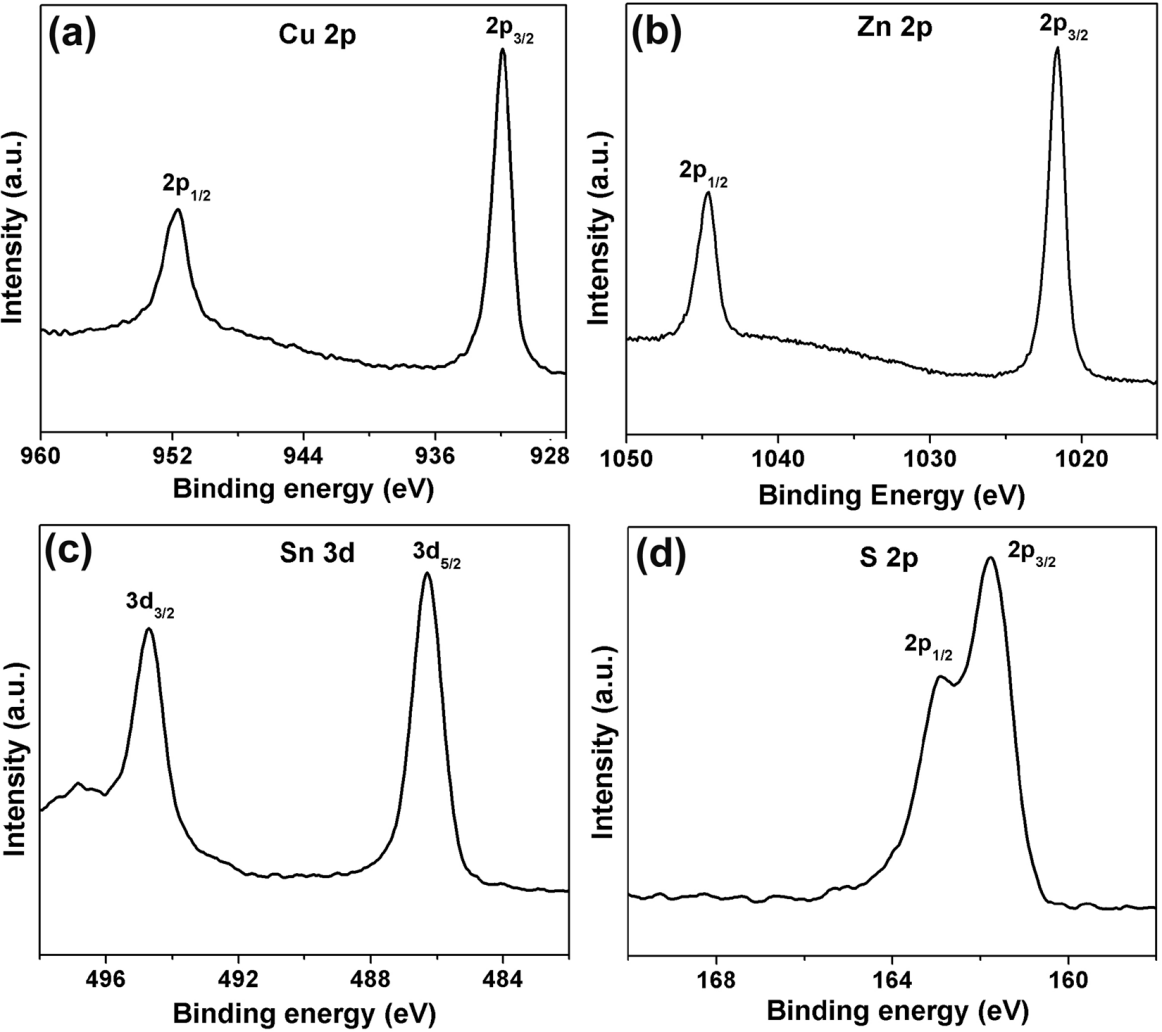
High-resolution XPS spectra of (**a**) Cu, (**b**) Zn, (**c**) Sn, and (**d**) S for wurtzite CZTS-NS.

X-ray photoelectron spectroscopy (XPS, Fig. 3) has been performed to confirm the oxidation states of the four elements in CZTS-NS. The qualitative XPS survey spectrum for CZTS-NS confirms the presence of Cu, Zn, Sn, S, C, and O (see Supplementary Fig. S2). High-resolution XPS spectra of Cu 2*p*, Zn 2*p*, Sn 3*d*, and S 2*p* have been measured to determine the oxidation states of each element, respectively (Fig. 3a–d). The peak of Cu 2*p* appears at 931.9 eV (2*p*_3/2_) and 951.7 eV (2*p*_1/2_), which in accordance with the value of Cu (I). The Zn (II) state is confirmed by peaks located at 1021.6 eV and 1044.7 eV with its characteristic peak separation of 23.1 eV. The peak of Sn 3*d* appears at binding energies of 486.3 and 494.7 eV, which can be assigned to Sn (IV) with a peak separation of 8.4 eV. The sulfur spectrum can be assigned to the presence of sulfide ion at binding energies of 161.8 and 163.0 eV with a doublet separation of 1.2 eV. The peak positions of Cu 2*p*, Zn 2*p*, Sn 3*d*, and S 2*p* are in good agreement with literature reports^27^.

The CZTS-NS when dispersed in hexane and ultrasonicated for 10 min forms a dark color solution, which indicates a strong absorption at visible wavelengths. The optical properties of as-synthesized CZTS-NS have been studied by UV-Vis absorption spectroscopy (see Supplementary Fig. S3). The wurtzite CZTS-NS exhibit board absorption in the visible region. The band gap of wurtzite CZTS is calculated by plotting (*Ahv*)^2^ versus *hv* (*A* = absorpance, *h* = Planck’s constant and *v* = frequency) and extrapolating the linear portion of the spectrum in the band edge region. The band gap for CZTS-NS is determined to be 1.54 ± 0.25 eV, in good accordance with those previously reported for nanoparticles synthesized by solution methods^27,28^.

### Formation mechanism of CZTS nanosheets

**Figure 4:**
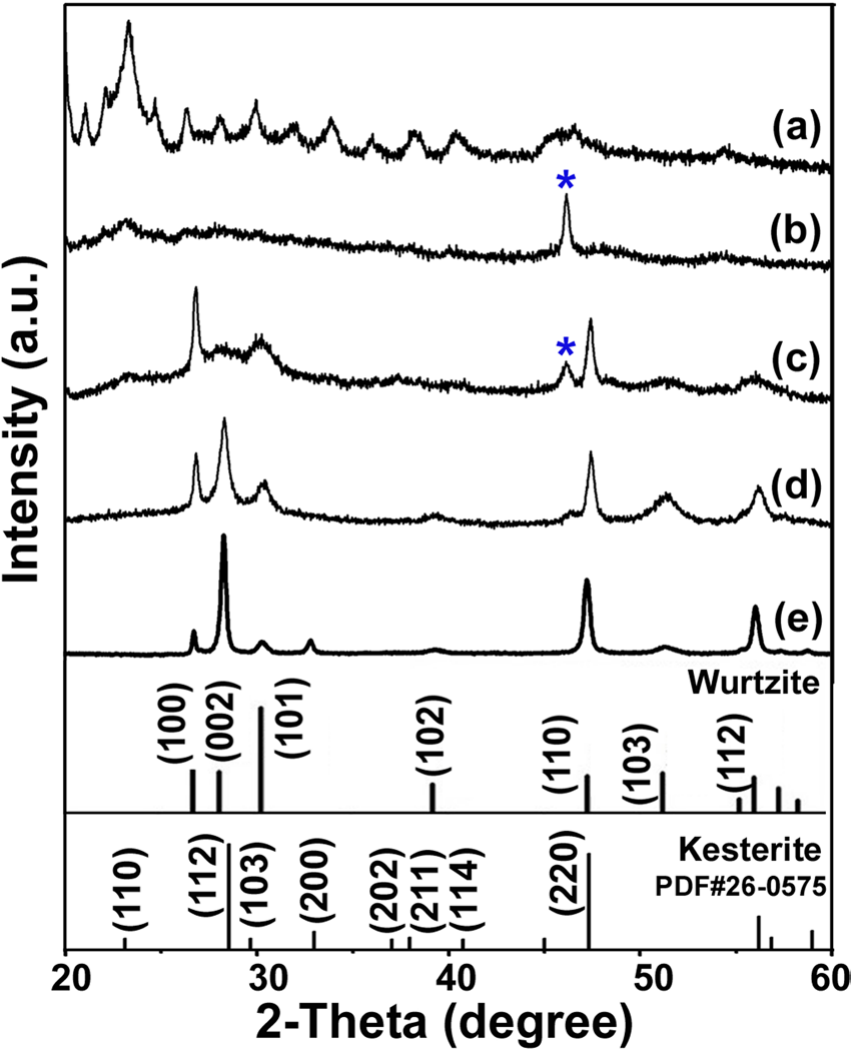
XRD patterns of wurtzite CZTS-NS synthesized at (**a**) 200, (**b**) 220, (**c**) 240, (**d**) 250, and (**e**) 270 ^o^C. The peaks marked with star (*) correspond to Cu_2−x_S.

During the synthesis of the wurtzite CZTS-NS, we found that the reaction temperature plays a vital role for the crystal phase and morphology control. Figure 4 shows the XRD patterns of intermediate products formed at 200, 220, 240, 250, and 270 °C during the synthesis of CZTS-NS. Amorphous particles are obtained at the initial stage at 200 °C (Fig. 4a). The amorphous particles with sizes of ~1–2 μm tend to accumulate together with small humps on the surface (see Supplementary Fig. S4a). During the heating-up process of the reaction system, an intermediate state of Cu_2−x_S binary phase is observed at 220 °C with an intense peak at 46.2 ° in the XRD pattern (Fig. 4b). Meanwhile, the amorphous particles gradually decompose and a small amount of triangular-shaped crystals being formed as shown in Supplementary Fig. S4b, which may correspond to the Cu_2−x_S nanocrystals. With the increase of reaction temperature to 240 °C (Fig. 4c), a combination of Cu_2−x_S and CZTS products are formed that may be due to the further reaction of Zn and Sn with the intermediate Cu_2−x_S binary phase. Meanwhile, accumulation of nanosheets initially is observed at this stage as shown in Supplementary Fig. S4c. Wurtzite CZTS is the predominant products when reaction temperature reaches up to 250 °C (Fig. 4d) and pure wurtzite CZTS nanocrystals can be obtained at 250 °C with reaction duration of 25 min (Fig. 1b). When the reaction temperature further increases to 270 °C (Fig. 4e), the crystallinity of wurtzite CZTS nanocrystals is improved, and the crystal structure is partially converted to kesterite structure as has been observed for other wurtzite-structured nanocrystals^28,49^. With further increase of reaction temperature, the thickness of nanosheets increases (see Supplementary Fig. S4d,e). We can conclude that the formation of CZTS-NS mainly involves three stages. Firstly, the amorphous precursor with micrometer size initially forms in the solvent. In the second stage, the precursor decomposes to form Cu_2−x_S nuclei. Finally, Zn and Sn incorporate into the Cu_2−x_S nanocrystals together with further decomposition of the precursor and the CZTS-NS are formed. Detailed investigation of the formation mechanism is ongoing.

### Characterization of CZTS thin films and photovoltaic measurements

**Figure 5:**
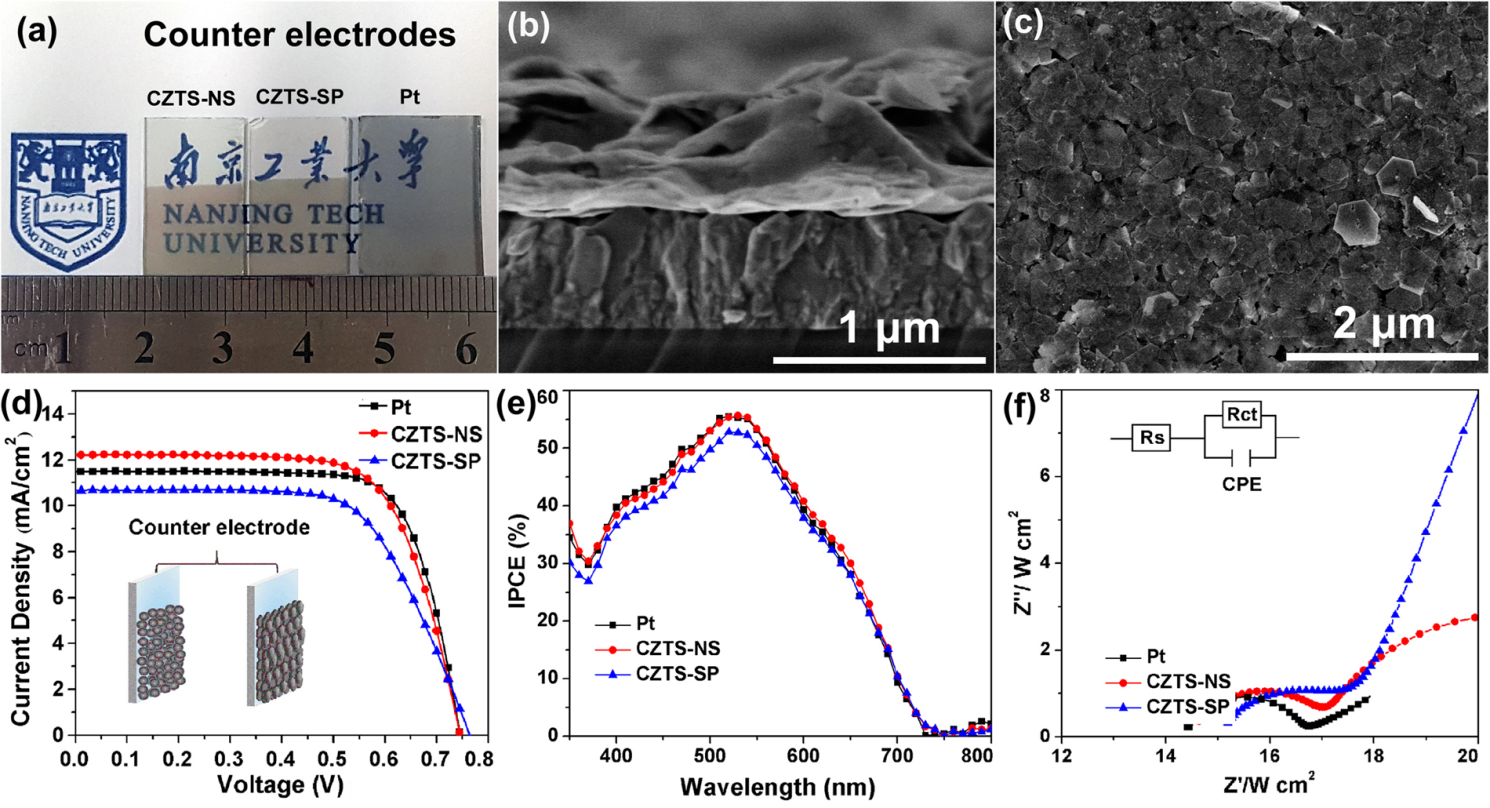
(**a**) Photograph of transparent CZTS-NS, CZTS-SP, and Pt thin films on FTO glass, (**b**) cross-section and (**c**) surface SEM images of CZTS-NS thin film made by spray painting method, (**d**) current-voltage curves and (**e**) incident photon-to-electron conversion efficiency (IPCE) of DSSCs fabricated using the CZTS-NS, CZTS-SP, and Pt thin films as counter electrodes (CEs), and (**f**) Nyquist plots from the symmetric cells with two identical CEs of the CZTS-NS, CZTS-SP, and Pt thin films. The cells were measured with a frequency range from 100 MHz to 1 Hz. The inset of (**f**) shows the corresponding equivalent circuit diagram. The inset of (**d**) is an illustration of CEs composing of the CZTS-NS and CZTS-SP thin films.

The photoelectrochemical property has been investigated by using as-prepared CZTS-NS as the counter electrode for DSSCs. We have chosen phase-pure wurtzite CZTS-NS prepared at 250 °C for 25 min to be used as counter electrode. It is well known that the optical, electrical, and catalytic properties are generally dependent on the morphology of the nanocrystals. In order to investigate the influence of (002) facets on the catalytic activity of CZTS, CZTS nanospheres (CZTS-SP) without anisotropic growth (see Supplementary Fig. S5) have been synthesized by a modified procedure for comparison, as described in the experimental section. Figure 5a shows optical images of CZTS-NS, CZTS-SP, and Pt thin films on FTO glass, with all appearing semi-transparent. The Pt counter electrode has been prepared in-house by DC sputtering (Leica EM SCD 500) and the thickness is controlled to be 5 nm using a quartz crystal film thickness monitoring system (Leica EM QSG 100). From the cross-section SEM image of CZTS-NS thin film (Fig. 5b), CZTS thin film with optimized thickness of only ~30–50 nm is obtained, which is much thinner than CZTS CEs reported previously^21,22,24^. As the CE materials should possess both high electrocatalytic activity and electrical conductivity^6^, CE films that are too thick will result in high resistance and lower *J*_sc_^24^. The large surface area of CZTS-NS can also decrease the amount of CZTS nanocrystals needed for the catalytic reaction. The surface of the thin film is densely packed nanosheets without any obvious cracks (Fig. 5c).

Figure 5d,e demonstrate the current density-voltage (*J-V*) and incident photon-to-electron conversion efficiency (IPCE) curves of DSSCs using Pt, CZTS-NS, and CZTS-SP CEs, respectively. The corresponding characterisitic parameters, such as short-circuit photocurrent density (*J*_sc_), open-circuit voltage (*V*_oc_), fill factor (FF), and power conversion efficiency (*η*) are summarized in Table 1. As shown in Fig. 5d, 5.58% efficiency is achieved when the CZTS-SP is used as CE in DSSCs, which is mainly due to the ratively low *J*_sc_ of 11.52 mA·cm^−2^ and FF of 61.99%. When the CZTS-NS is used as CE for DSSCs, the efficiency increases by 20% with *J*_sc_ of 12.21 mA·cm^−2^, *V*_oc_ of 0.74 V, FF of 69.61%, and efficiency of 6.68%. The efficiency is comparable to that of DSSCs with Pt CE (*η* = 6.70%, *J*_sc_ = 11.50 mA·cm^−2^, and *V*_oc_ = 0.74 V). We have summarized the CZTS CEs prepared by solution-based methods (hot-injection^24,50,51^, spin coating^52,53^, sol-gel^54^, SILAR^55^, and solvothermal^21,22,56^), and vacuum-based method^32,57^ (pulsed laser deposition, sputtering, *etc*.) in Supplementary Table S1. We note that this is the highest reported efficiency of CZTS CE without any selenization or high temperature (>500 °C) annealing process using colloidal chemistry method. Although the efficiency using CZTS-NS CE is not as high as CZTS CE obtained by other methods, our study provides a simple and promising way to achieve low-cost CE with enhanced performance via crystal facet engineering when using 2D CZTS-NS. The IPCE value (Fig. 5e) of the CZTS-NS is very close to that of Pt, and much higher than that of CZTS-SP, which is in accordance with the I-V results, indicating that the catalytic behavior of CZTS-NS is similar to that of Pt to reduce oxidized triiodide (I_3_^−^) to iodide (I^−^), but much better than that of CZTS spherical particles.

**Table 1:** Photovoltaic parameters of DSSCs with different CEs and the simulated data from EIS spectra.

CEs	*V*_oc_ (V)	*J*_sc_ (mA/cm^2^)	FF (%)	Eff. (%)	*R*_s_(Ω·cm^2^)	*R*_ct_ (Ω·cm^2^)
CZTS-NS	0.74	12.21	69.61	6.68	14.47	2.55
CZTS-SP	0.74	11.52	61.99	5.58	15.02	3.09
Pt	0.74	11.50	74.11	6.70	14.34	2.32

Abbreviations: *V*_oc_: open-circuit voltage; *J*_sc_: short-circuit photocurrent density; FF: fill factor; Eff.: energy-conversion efficiency; *R*_s_: series resistance; *R*_ct_: charge-transfer resistance. All measurements are taken under AM 1.5 G (100 mW/cm^−2^) sunlight illumination.

### Electrochemical impedance spectroscopy

Electrochemical impedance spectroscopy (EIS) measurements and cyclic voltammetry (CV) are performed to investigate the electrochemical and catalytic activity of various CEs. For the EIS measurements, symmetric cells are fabricated with two identical electrodes (CE//electrolyte//CE). Figure 5f shows the Nyquist plots for illustrating the impedance characteristics. The plots are further fitted with an equivalent circuit, as shown in the inset of Fig. 5f. The high-frequency intercept on the real axis (*Z*’ axs) represents the series resistance (*R*_s_), which is mainly composed of the bulk resistance of CZTS, the resistance of fluorine-doped SnO_2_ (FTO) glass, and the contact resistance. The semicircle in the high-frequency range arises from the charge-transfer resistance (*R*_ct_) and the corresponding constant phase angle element (CPE) at the electrolyte/counter electrode interface. The semicircle in the low-frequency range represents the Nernst diffusion impedance of the triiodide/iodide couple in the electrolyte^24^. The values of *R*_s_ and *R*_ct_ obtained by fitting the spectra with an EIS analyzer are summarized in Table 1. The *R*_s_ values of CZTS-NS, CZTS-SP, and Pt are 14.47, 15.02, and 14.34 Ω·cm^2^, respectively, which indicates comparable conductivity for CZTS-NS and Pt as CE material. As compared to CZTS-SP, the smaller *R*_s_ value for CZTS-NS can be explained by better connectivity with the nanosheets which can facilitate electron transfer. The *R*_ct_ of CEs is a major factor in the performance of DSSCs, suggesting a catalytic activity for triiodide reduction. The similar *R*_ct_ values for CZTS-NS (2.55 Ω·cm^2^) and Pt (2.32 Ω·cm^2^) electrodes may be an important factor for comparable energy conversion efficiency of DSSCs. Simultaneously, the *R*_ct_ value for the CZTS-NS is much smaller than that of the CZTS-SP, indicating the obvious excellent electrocatalytic property for oriented growth of the CZTS-NS with highly exposed (002) facets.

### Catalytic activity measurement

**Figure 6:**
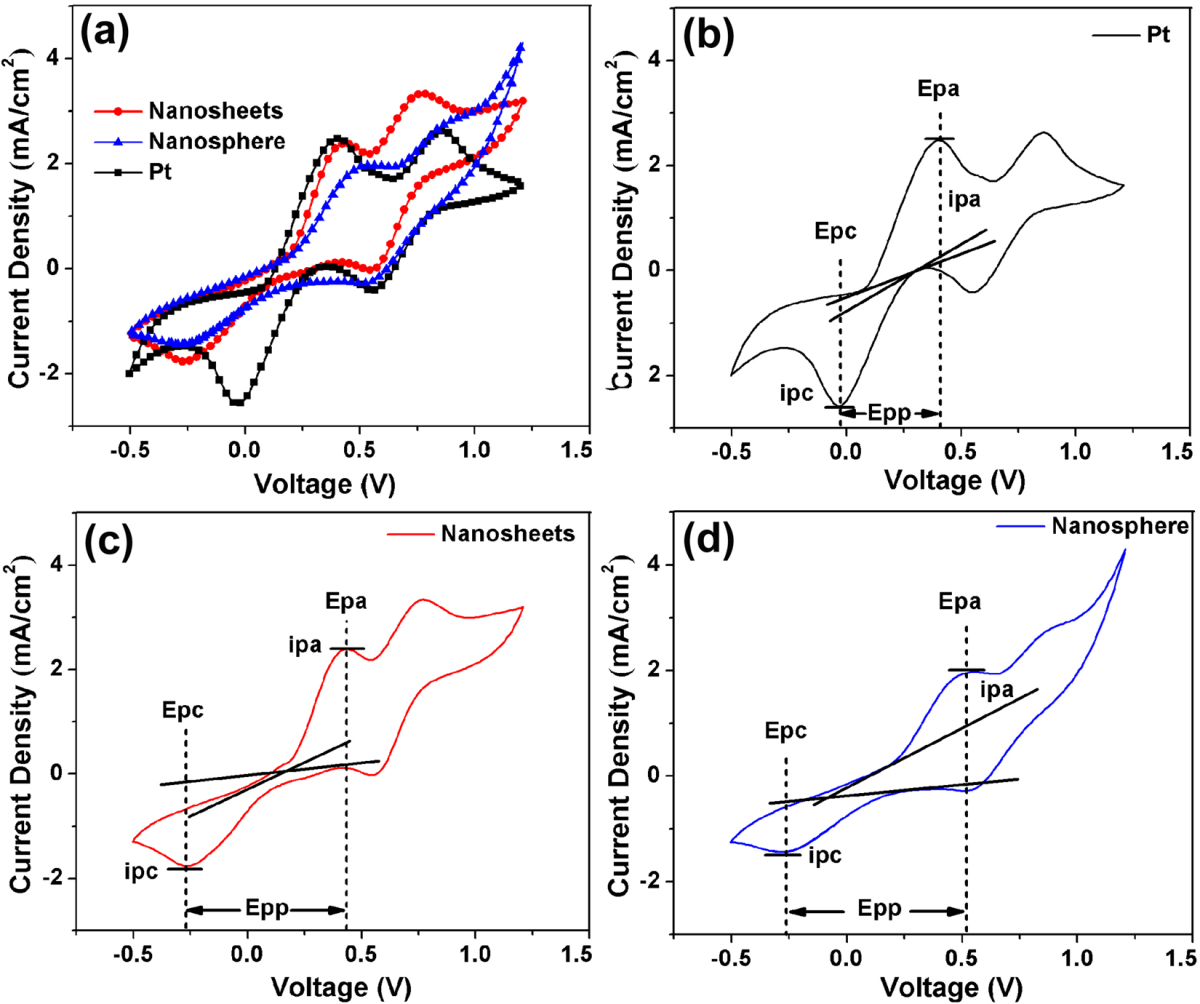
(**a**) Cyclic voltammograms for various CEs of the CZTS-NS, CZTS-SP, and Pt electrodes with a scan rate of 50 mV·s^−1^. (**b**–**d**) The peak current (*i*_p_), peak voltage (*E*_p_), and peak to peak separation (*E*_pp_) of different CEs.

Cyclic voltammetry (CV) measurement has been performed using a three-electrode system comprising the working electrode, a Pt foil counter electrode, and an Ag/AgCl reference electrode. Figure 6a shows the CV curves of CZTS-NS, CZTS-SP, and Pt working electrodes, respectively, with a scan rate of 50 mV·s^−1^. Different electrodes have shown similar CV curves with two pairs of redox peaks (Ox-1/Red-1, Ox-2/Red-2) being observed. As the reaction of I^−^/I_3_^−^ redox electrolyte in CEs is I_3_^−^ + 2e^−^ ↔ 3I^−^, so the reduction process is pertinent to the CEs performance and should be considered in our analysis^58^. Two important parameters for catalytic activity are the peak current (*i*_p_) and the peak-to-peak separation (*E*_pp_)^59–61^. As shown in Fig. 6b, the cathodic peak current density (*i*_pa_) and anodic peak current density (*i*_pc_) of CZTS-NS are 1.82 mA·cm^−2^ and 1.73 mA·cm^−2^, higher than that of CZTS-SP (*i*_pa_ = 1.21 mA·cm^−2^, *i*_pc_ = 1.18 mA·cm^−2^), but slightly lower than that of Pt (*i*_pa_ = 2.14 mA·cm^−2^, *i*_pc_ = 2.15 mA·cm^−2^). The *E*_pp_ in a pair is in an inverse correlation with the standard electrochemical rate constant of the corresponding redox reaction^58^. The lower *E*_pp_ represents the higher kinetic ability for I_3_^−^ reduction^57^. It is observed that the *E*_pp_ of CZTS-NS, CZTS-SP, and Pt is 0.66 V, 0.73 V, and 0.44 V, respectively, indicating the electrochemical rate in the sequence of Pt > CZTS-NS > CZTS-SP. The comparatively slower electrochemical rate for CZTS based electrode can be ascribed to the thicker CZTS thin films than Pt on FTO glass^58^. Furthermore, Pt should provide better conductivity than semiconducting CZTS nanocrystals. The high peak current density and lower *E*_pp_ value indicate that the catalytic activity of CZTS-NS is superior to CZTS-SP, in accordance with the EIS values above. Both EIS and CV results indicate that CZTS-NS provide comparable electrocatalytic activity as Pt, and the (002) facets of CZTS-NS have superior catalytic activities.

## Discussion

The physical and chemical properties of nanomaterials are strongly influenced by the microstructural parameters such as crystal phase, morphology, size, *etc*. We have carried out theoretical calculations to investigate the related properties of CZTS. The space group of wurtzite structured CZTS is *P6*_3_ *mc* with 3.843 Å in-plane (*xy* plane) and 6.337 Å *c*-plane experimental lattice constants. The wurtzite structure CZTS is made on the basis of parent material AS_4_, where A is occupied by 50% Cu, 25% Zn, and 25% Sn. Therefore, a 2 × 2 × 1 supercell is used to simulate the crystal structure and calculate several possible arrangements (see Supplementary Fig. S6). We relax the ions until the Hellmann-Feynman forces are less than 0.01 eV·Å^−1^. The relaxation results are listed in Supplementary Table S2 and the most stable structure are configurations 3 and 4 in which one Zn atom, one Sn atom, and two Cu atoms are bonded to per S atom. Our calculated in-plane and *c*-plane lattice parameters are 7.563 Å and 6.249 Å, respectively, which are slightly smaller than the experimental value.

**Figure 7:**
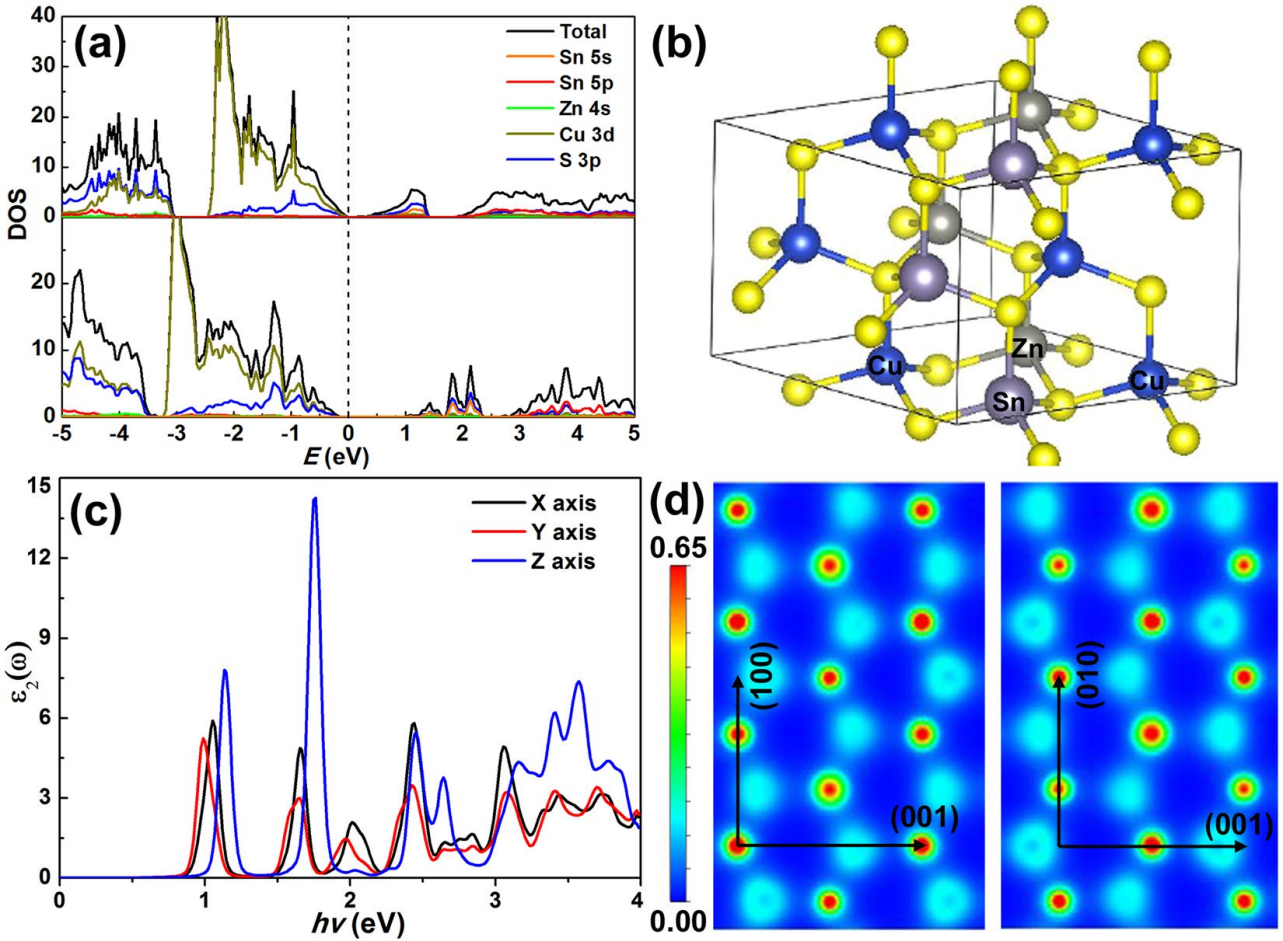
(**a**) DOS of wurtzite CZTS for GGA (top) and HSE06 (bottom) method. (**b**) Crystal structure of wurtzite CZTS for calculation. (**c**) The calculated imaginary dielectric spectra along the different direction. (**d**) The electric charge density of wurtzite CZTS.

The calculated density of states (DOS) is shown in Fig. 7a. As expected from generalized gradient approximation (GGA)  calculation (Fig. 7a, top), wurtzite CZTS is a semiconductor with a gap about 0.1 eV. Generally, density functional theory (DFT) tends to systematically underestimate the band gap, but the resulting dispersion of individual bands is less problematic. Other methods incorporating many-body corrections, such as hybrid functional^62^, GW approximation^63^, can correct the systematic DFT band gap underestimation and provide good agreement with experiment. Therefore, a calculation with hybrid exchange-correlation functional (HSE06) is also performed and the result is shown in Fig. 7a (bottom). The calculated gap with HSE06 is about 1.2 eV, which is slightly smaller than that for kesterite CZTS^64^. The upper valence band (VB) is mainly composed of the antibonding states between Cu 3d and S 3p, while Sn 5 s and S 3p primarily make contributions to the bottom conduction band (CB).

The calculated imaginary dielectric spectra along the different direction are shown in Fig. 7c. It is seen that wurtzite CZTS dielectric tensor *ε*(ω) exhibits strong anisotropy with respect to the relative orientation of the crystal lattice, which leads to the non-diagonal dielectric tensor. Concretely, the imaginary dielectric spectra along the *x* and *y* directions of lattice vector have analogous behavior but differ from the vertical direction. From Fig. 7c, we conclude that the sample would have strong absorption for blue light along the *z* axis at energy of ~1.7 eV around the band gap of wurtzite CZTS. From Fig. 7d, we also find charge transfer along the [001] direction.

Next we turn to pay attention to the surface of wurtzite CZTS. To determine the stable crystal plane, the surface energies are estimated by taking the energy difference between the relaxed slabs and the bulk structure with the same number (*n*) Cu_2_ZnSnS_4_ units as the slab, then the corresponding surface energies (*γ*) is given by Equation (1):

1 $$\gamma =\frac{{E}_{s}-n{E}_{b}}{2S}$$

where *E*_s_ is the total energy of the slab, *E*_*b*_ is the energy of bulk structure and *S* is the surface area of the slab. To maintain the same stoichiometry in the slab compared to the bulk, we constructed the available terminations for (002), (010), (100), (101) and (1–10) surfaces in wurtzite CZTS (see Supplementary Fig. S7). Due to lack of van der Waals bond in CZTS, all the surface will produce dangling bonds. Thus breaking the weakest bonds parallel to a given crystal orientation will possess the lowest surface energy. Our calculation shows that, as expected, the energies for (002), (010), (100), (101) and (1–10) surface, are 31.24, 36.89, 21.48, 47.69 and 38.84 meV/Å^2^, respectively. This indicates that the most-abundant surface in wurtzite CZTS sample is the (100) surface, which has the lowest surface energy. The (002) facets have higher surface energy than the (001) facets. It is well known that high-index facets of nanocrystals usually possess high surface energy and a high density of atomic steps, ledges, and kinks, which enable them to have high activity, thus promoting their potential applications as highly efficient catalysts^11,29,65,66^. The calculation is in good accordance with literature reports and our experimental results, which confirm that CZTS-NS with high energy (002) facets exhibits better photocatalytic activity. Interestingly, the (100) surface and (010) surface are symmetrical in *P6*_3_ *mc* space group, but with disparate surface energy. The main reason is that we need to break the S-Cu and S-Zn bonds in (100) surface and S-Cu and S-Sn bonds in (010) surface, while, S-Sn bonds are stronger than S-Zn’s when considering the surface energy.

As stronger reducibility for I_3_^−^ molecule has been observed for the (002) facets of wurtzite CZTS-NS, the adsorption energy between the I_3_^−^ ions and (002) surface has been investigated in order to explain this phenomenon. To simulate the real experimental environment, the (002) surface with a thickness of 2 nm has been constructed in our calculation. The absorption energies (*E*_*b*_) are defined as:

2 $${E}_{b}={E}_{{I}_{3}^{-}-C{\rm{Z}}{\rm{T}}S}-({E}_{{I}_{3}^{-}}+{E}_{C{\rm{Z}}{\rm{T}}S})$$

where *E*_I__3_^−^−CZT represents the total energy of the I_3_^−^ and (002) surface of wurtzite CZTS; *E*_I__3_^−^ is the total energy of the isolated I_3_^−^ molecule; and *E*_*CZTS*_ represent the total energy of (002) surface. To determine the energetically preferred adsorption, the I_3_^−^ molecule is placed at three different initial positions (absorbed on Cu, Sn and Zn atoms) on the (002) surface, and after full relaxation, the converged structures are obtained (see Supplementary Fig. S8). The calculated adsorption energies of I_3_^−^ on (002) surface of Cu, Sn and Zn atoms are −0.82, −0.64, and −0.70 eV, respectively. The results indicate the strong interaction between I_3_^−^ and the (002) surface. For I_3_^−^ molecule adsorbed on Cu atoms of (002) surface, the original bond length of I_1_ − I_2_ in the free I_3_^−^ complex is 0.268 nm, which increases to 0.282 nm on adsorption to the (002) surface. In short, our calculation indicates that the (002) surface has remarkable reducibility for I_3_^−^ ions, which can be explained by the electron clouds interaction between I_1_ atom and (002) facets^67^.

According to the above analysis, we hypothesize the excellent photoelectrochemical performance of CZTS-NS as a Pt-free CE based on the following aspects. First, the nanosheet networks of CZTS-NS can not only provide large active catalytic surface area to adsorb and catalyze I_3_^−^ but also facilitate electron transfer. Second, high energy (002) facets of CZTS-NS have remarkable reducibility for I_3_^−^ ions, which enahnces the photoelectrochemical activity. Finally, the obtained wurtzite structure provides high carrier concentration and low resistivity^39^, which can accelerate the transportation of photogenerated electron at the electrode/redox electrolyte interface. Furthermore, post-synthetic ligand exchange can efficiently remove the insulating organic molecules to increase the conductivity.

## Conclusions

In summary, we have demonstrated a simple one-pot colloidal chemistry method to synthesize wurtzite-structured 2D CZTS-NS with high-energy (002) facets exposed. The formation of CZTS-NS involves the initial Cu_2−x_S nuclei and further incorporation of Zn and Sn. A simple and scalable spray painting method has been utilized to fabricate high quality CZTS thin film CE. DSSCs using CE composed of CZTS-NC of only ~30–50 nm thick without selenization or high temperature annealing treatment have exhibited power conversion efficiency of 6.68%, which is comparable to Pt (6.70%), but obviously higher than that based on the 0D CZTS nanospheres (5.58%). The enhancement of photoelectrochemical activity for CZTS-NS can be ascribed to the large active catalytic surface area, remarkable reducibility for I_3_^−^ ions with high energy (002) facets, and the rapid transportation of photo-generated electrons in the wurtzite structure. Theoretical calculations also indicate high surface energy for (002) facets and remarkable reducibility of I_3_^−^ ions for wurtzite CZTS-NS. This work will likely pave a new way to explore low-cost 2D sulfide nanomateials for solar cells, photocatalysis, and energy storage applications.

## Methods

### Materials

Copper(II) acetylacetonate (Cu(acac)_2_, Aladdin, 97%), zinc acetylacetonate (Zn(acac)_2_, Aladdin, 97%), tin(IV) chloride pentahydrate (SnCl_4_·5H_2_O, 99%), 1-dodecanethiol (DDT, Aladdin, 98%), oleylamine (OLA, Aladdin, 80~90%), formamide (CH_3_NO, Lingfeng Chemical Reagent, 99%), and ammonium sulfide ((NH_4_)_2_S, Tongya Chemical) were used without further purification. Other chemicals were all of analytical grade.

### Synthesis of wurtzite CZTS nanosheets (CZTS-NS)

All the experiments were carried out in a fume hood under inert N_2_ atmosphere using standard Schlenk techniques. In a typical synthesis of wurtzite CZTS-NS, 2 mmol Cu(acac)_2_, 1 mmol Zn(acac)_2_, and 1 mmol SnCl_4_·5H_2_O were added into a 100 mL four-neck round-bottom flask containing 20 mL 1-dodecanethiol. The mixture was stirred at room temperature for 30 min with nitrogen purging and then gradually heated to 120 °C and kept for 20 min. After that, the solution was heated to 200 °C and maintained until it turned brown, and then the reaction temperature was raised to 250 °C at the rate of 2 °C/min and held at this temperature for 25 min. After reaction, the mixture was cooled down to room temperature and the product precipitated and washed with hexane for three times. In order to determine the formation mechanism of CZTS-NS, the intermediate products formed at different reaction temperatures (200, 220, 240, 250, and 270 °C) were extracted for further measurements.

### Synthesis of wurtzite CZTS nanospheres (CZTS-SP)

The synthesis process of wurtzite CZTS-SP was the same as CZTS-NS except for the changing of reaction solvent and reaction time. The solvent was a mixture of 12 mL oleylamine and 18 mL 1-dodecanethiol with a volume ratio of 2:3. The reaction mix was held at 250 °C for 120 min.

### Ligand exchange with S^2−^

The ligand exchange was conducted according to the process reported before^47^. For a typical ligand exchange procedure, 150 mg CZTS-NS were dispersed in 6 mL hexane by ultrasonication for 10 min. 2 mL (NH_4_)_2_S (20% aqueous) was dissolved in 20 mL polar formamide (FA) with vigorous shaking to form a uniform dispersion. Then the above two solutions were mixed together and stirred for about 30 min, leading to a complete phase transfer of CZTS-NS from hexane to FA.

### Preparation of CZTS and Pt counter electrodes (CEs)

CZTS nanocrystals with ligand exchange were dispersed in ethanol and ultrasonicated for 10 min to form a uniform ink with a concentration of ~20 mg/mL. The ink was further diluted to provide different concentrations. CZTS thin films with various thicknesses were spray-deposited using suspension with different concentrations onto FTO glass, followed by annealing under Ar atmosphere at 300 °C for 30 min. Pt electrode (~5 nm) was prepared by DC sputtering of Pt on FTO glass in Ar atmosphere at deposition pressure of 10^−2^ Pa and current of 15 mA.

### Assembly of DSSCs

The TiO_2_ photoanodes with thickness of ~600 nm and active area of ~0.16 cm^2^ were purchased from OPV Tech Co., Ltd. The photoanodes were treated at 100 °C for 30 min in an oven. When the temperature decreased to 80 °C, they were immersed in 0.3 mM N719 solution with absolute ethyl alcohol and sensitized at 30 °C for 20 h. DSSCs were assembled with TiO_2_ photoanodes and CZTS/Pt CEs and sealed up by 50 μm thick PTFE Tape film to avoid short circuit.

### Characterization of materials

The crystal structure of the products was characterized using X-ray diffractometer (XRD, Rigaku-Smart Lab Advance) with Cu-Kα radiation (λ = 1.5408 Å) as the X-ray source. The morphology of the samples was studied using field-emission scanning electronic microscopy (FESEM, HITACHI S-4800) and transmission electron microscopy (TEM, JEOL JEM-2100) coupled with selected area electron diffraction (SAED) operating at 200 kV. Atomic force microscope (AFM, XE-100) was used to investigate the thickness of CZTS-NS. The Raman spectra of the samples were measured using a Raman spectrometer (Horiba Labram HR 800). The valence state and composition of CZTS samples were characterized by X-ray photoelectron spectroscopy (XPS, AXIS UltraDLD) and energy dispersive X-ray spectroscopy (EDX, equipped on the FESEM, HITACHI S-4800). The UV-Vis absorption spectra of CZTS were recorded on a UV-Vis spectrometer (Varian Cary 300). Fourier transforms infrared (FTIR) spectra were recorded using a Nicolet IS10 FT-IR spectrophotometer.

### Performance of solar cells

The photocurrent-voltage (J-V) measurements were taken on a Newport solar cell simulator (Oriel Sol2A) equipped with a 300 W Xenon lamp (light irradiation of 100 mW·cm^−2^). Incident photon-to-electron conversion efficiency (IPCE) spectra were recorded using an Oriel QE/IPCE Measurement Kit (equipped with 150–300 W full spectrum solar simulator).

### Electrochemical characterization

Electrochemical impedance spectroscopy (EIS) analysis was conducted in dummy cells by using an electrochemical workstation (AUTOLAB PGSTAT302N). The frequency scan was from 1 MHz to 1 Hz. Cyclic voltammetry (CV) was carried out in a three electrode system in an acetonitrile solution containing 0.1 M LiClO_4_, 0.01 M LiI, and 0.001 M I_2_ at a scan rate of 50 mV·s^−1^.

### Density functional theory (DFT) calculations

The DFT calculations have been performed using Vienna ab initio simulation package (VASP)^68,69^. The valence electrons including the semicore electrons in the case of Cu and Sn are treated with the projector augmented wave method^70^ and generalized gradient approximation of Perdew-Burke-Ernzerhof (GGA-PBE) are used with a cut-off energy of 500 eV for the expansion of the electronic wave function in the plane waves. For the structural relaxation and electronic structure calculations, the Brillouin-zone (BZ) integrations are performed with a Gaussian smearing of 0.05 eV over a 7 × 7 × 3 Monkhorst-Pack k-point mesh centered at ΓΓ. To calculate the surface energies for different crystal surface of wurtzite Cu_2_ZnSnS_4_ (CTZS), the slab model is used to simulate the surface with adding a vacuum layer of 15 Å.

### Data Availability

The datasets generated during and/or analyzed during the current study are available from the corresponding author on reasonable request.

## Electronic supplementary material

Supporting information
