# Supplementary material for: Synthesis of Wurtzite Cu2ZnSnS4 Nanosheets with Exposed High-Energy (002) Facets for Fabrication of Efficient Pt-Free Solar Cell Counter Electrodes
Source: Sci Rep. 2018 Jan 10;8:248. doi: 10.1038/s41598-017-18631-0 (PMC5762643; doi:10.1038/s41598-017-18631-0)
Supplement: Supplementary file 1 — Supporting information [file 41598_2017_18631_MOESM1_ESM.doc]

Supporting Information

Synthesis of Wurtzite Cu_2_ZnSnS_4_ Nanosheets with Exposed High-Energy (002) Facets for Fabrication of Efficient Pt-Free Solar Cell Counter Electrodes

Content

Figure S1---------------------------------------2

Figure S2---------------------------------------2

Figure S3---------------------------------------3

Figure S4---------------------------------------3

Figure S5---------------------------------------4

Figure S6---------------------------------------4

Figure S7---------------------------------------5

Figure S8---------------------------------------5

Table S1----------------------------------------6

Table S2----------------------------------------8

References--------------------------------------8


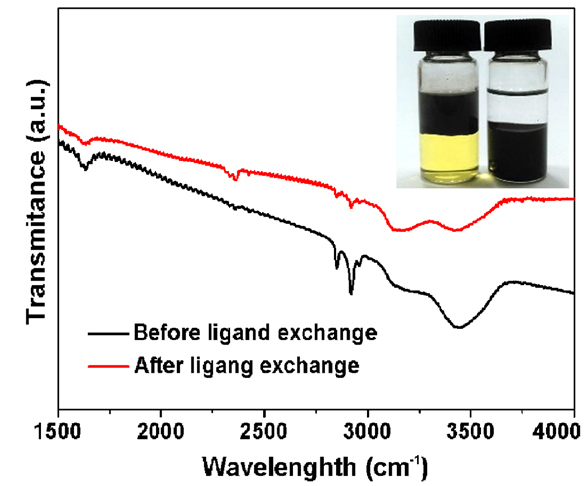


Figure S1. FTIR spectra of the CZTS-NS before and after ligand exchange. Inset shows the photograph of CZTS solutions before (left) and after (right) ligand exchange.


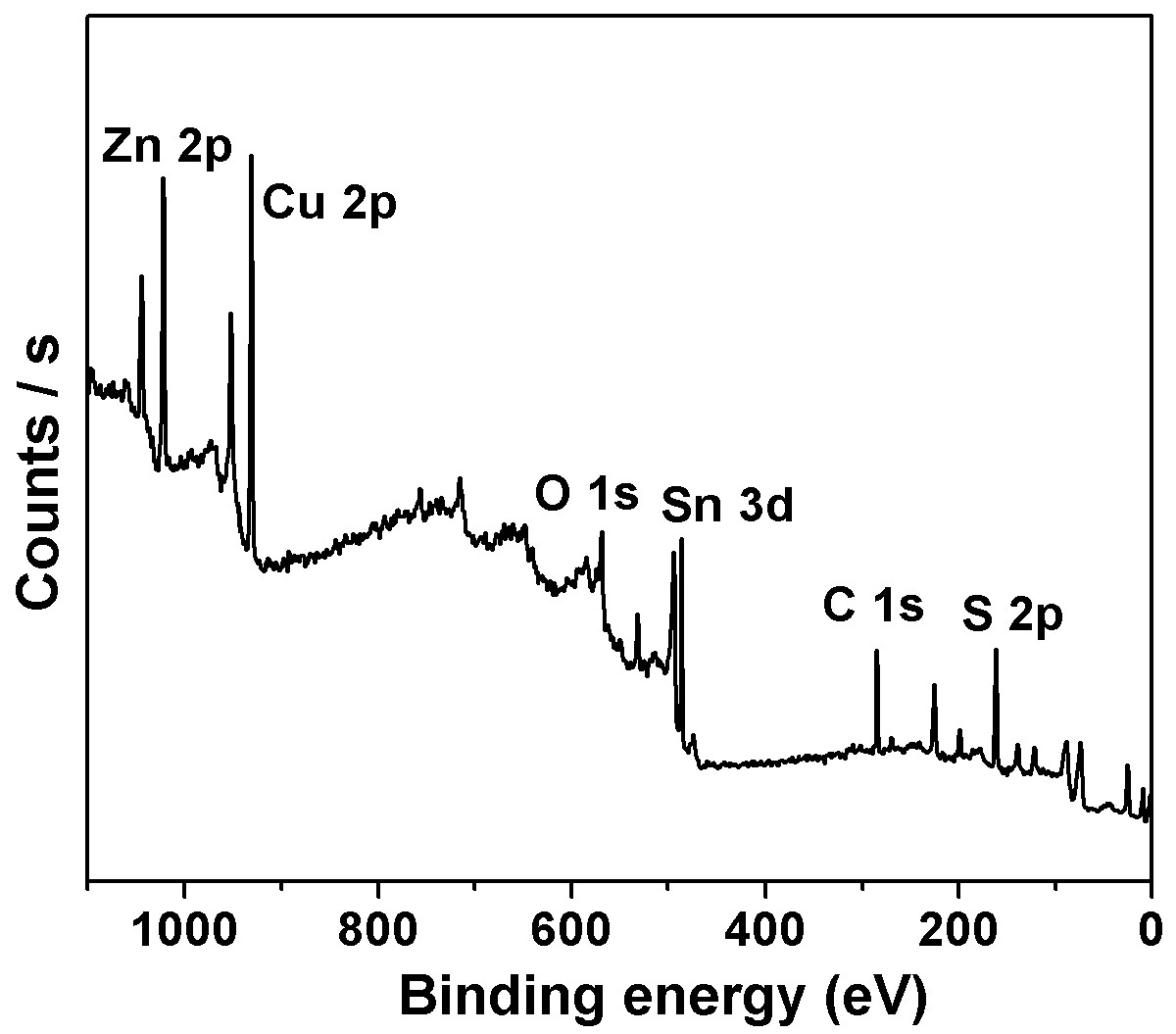


Figure S2. The qualitative XPS survey spectrum of the wurtzite CZTS-NS.


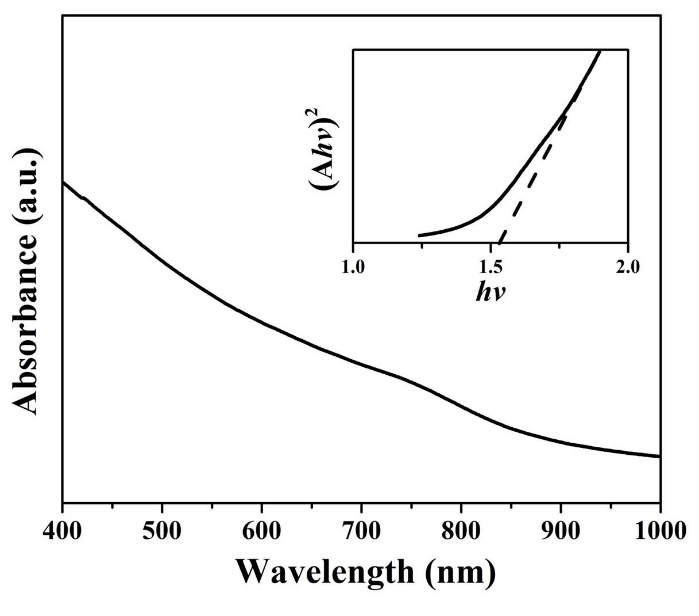


Figure S3. UV-Vis spectrum of synthesized CZTS nanosheets dispersed in hexane. Inset shows the plot of (Ahv)^2^ versus hv for determining the band gap.


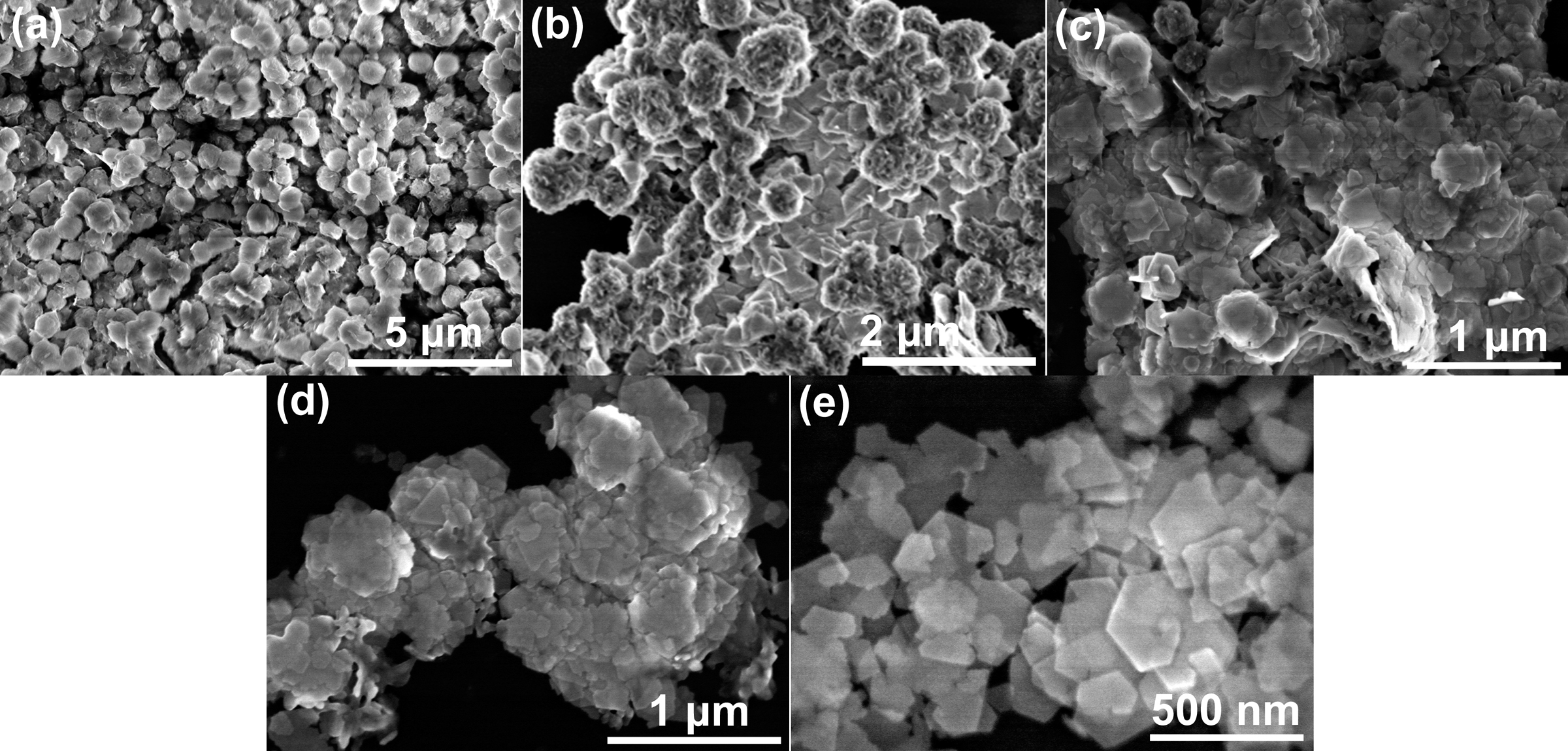


Figure S4. SEM images of wurtzite CZTS-NS synthesized at (a) 200, (a) 220, (a) 240, (a) 250, and 270 ^o^C.


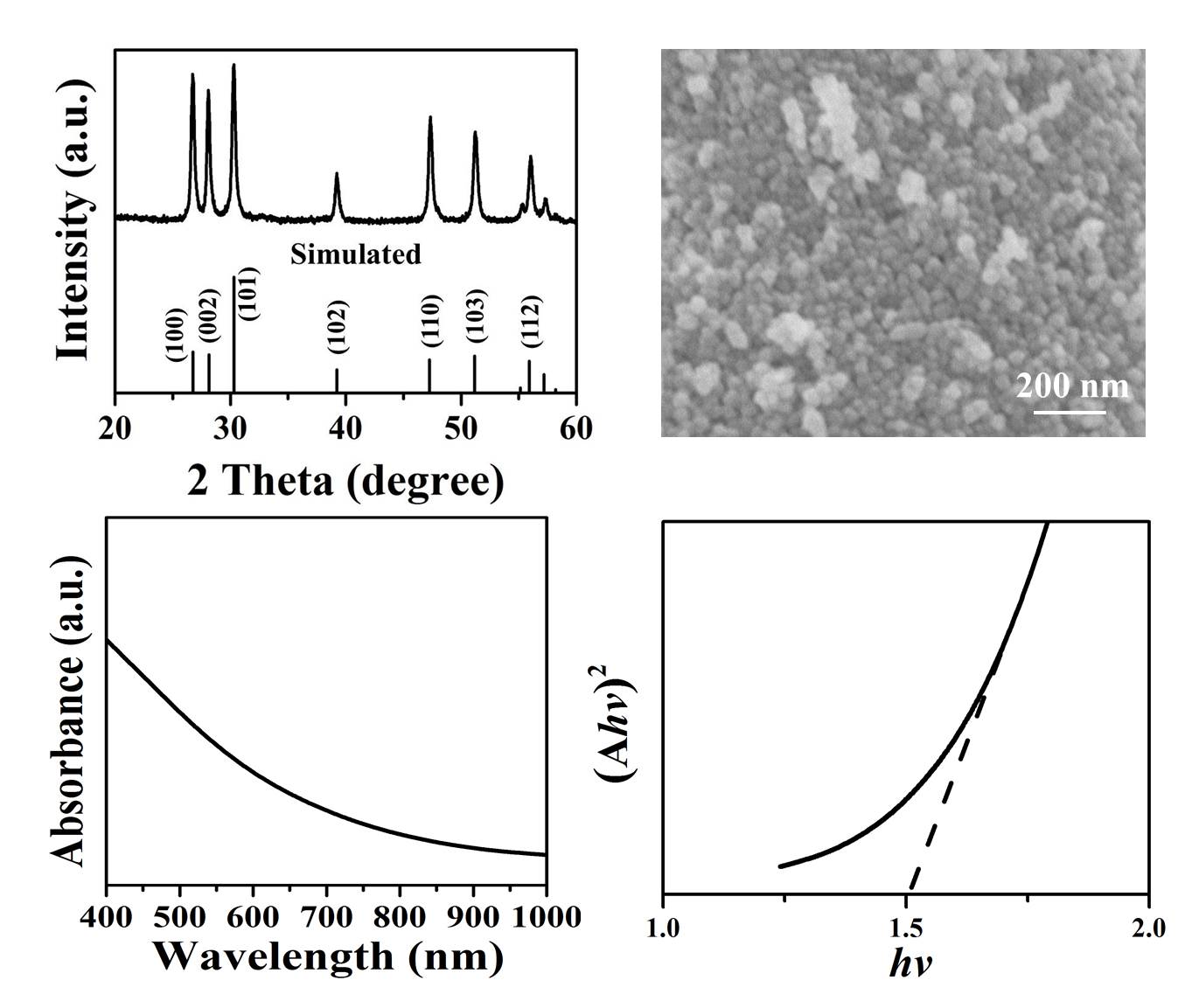


Figure S5. The XRD pattern, SEM image, UV-Vis spectra and plot of (Ahv)^2^ versus hv for determining the band gap of synthesized CZTS-SP.


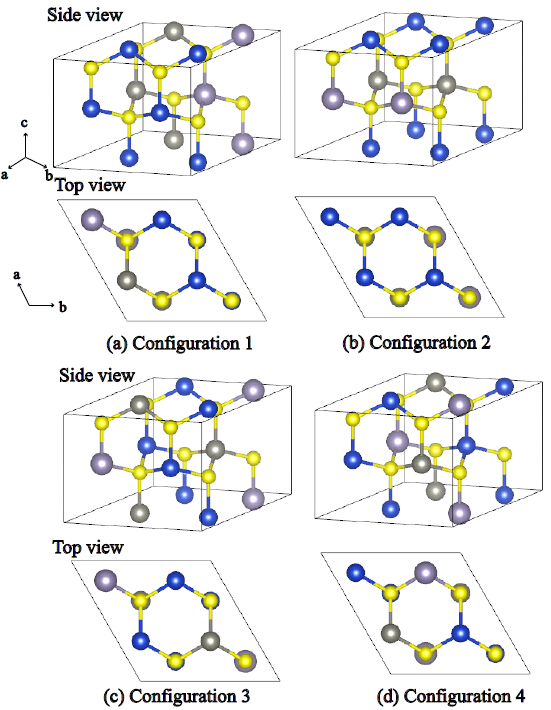


Figure S6. Several possible arrangements of crystal structure of CZTS.


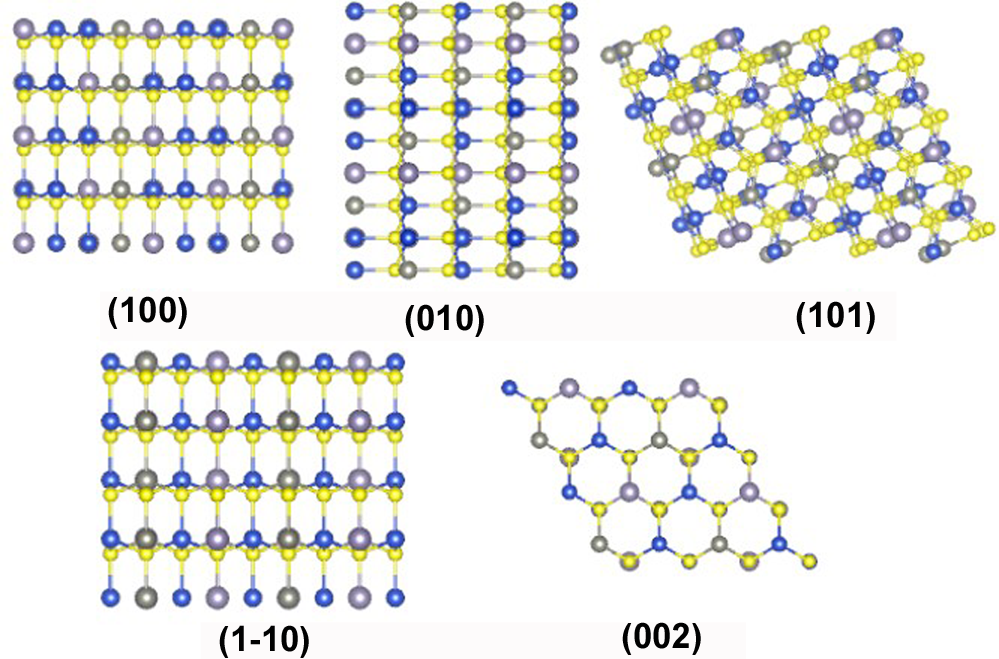


**Figure S7.** Models of different crystal surfaces of wurtzite CZTS.


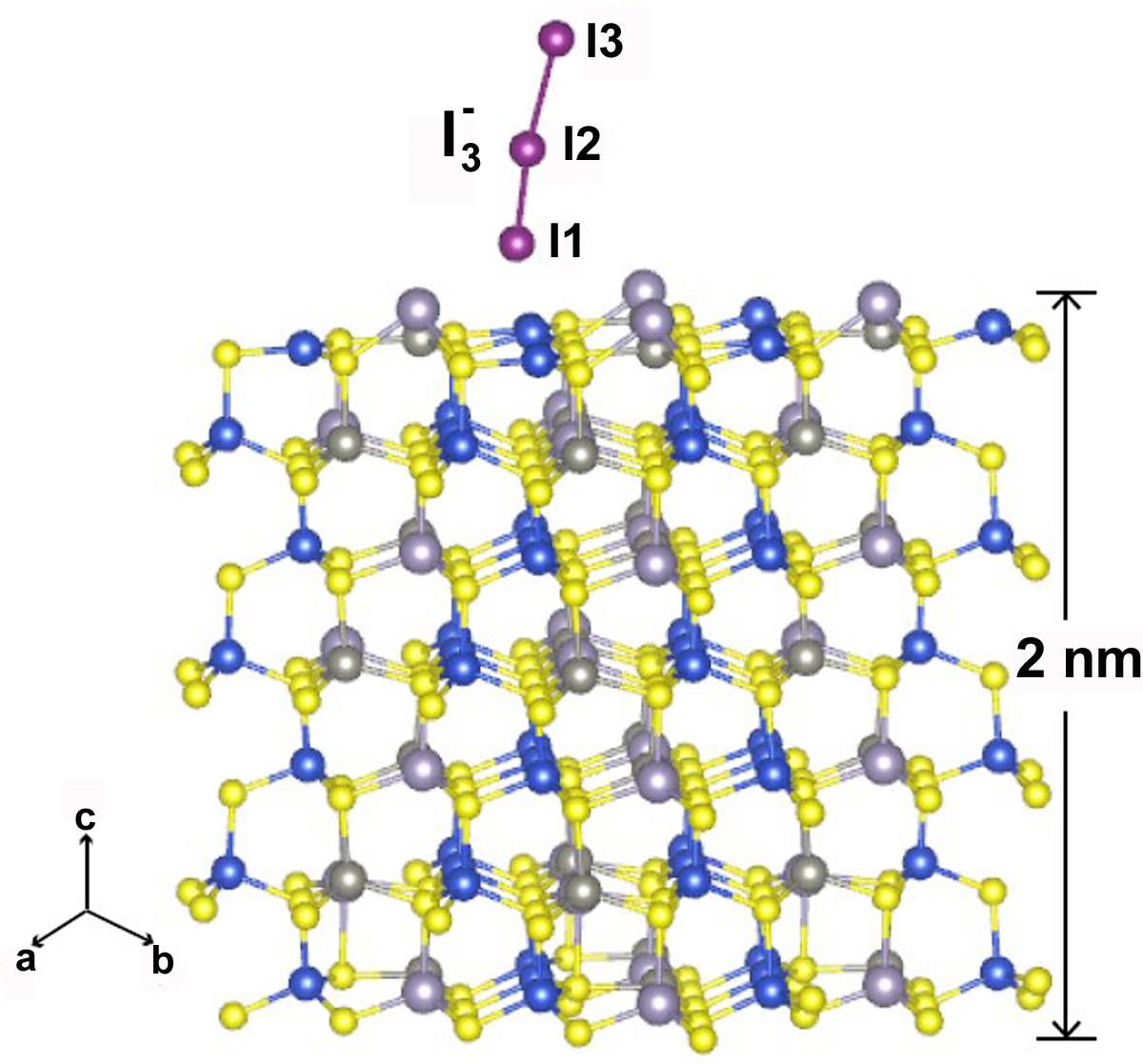


Figure S8. I^3-^ ions attached to the (002) facet of wurtzite CZTS.

Table S1 Summary of CZTS/Se-based CE for DSSCs

|  | CE | Crystal structure | Morphology/structure | Deposition method | Thickness | FF | PCE | PCE-Pt | Ref |
| --- | --- | --- | --- | --- | --- | --- | --- | --- | --- |
| Colloidal chemistry/hot injection--Particle | | | | | | | | | |
| 1 | CZTS | K | Particle | Spin coating | 180 nm | 40.3 | 3.62 |  | [1] |
|  | CZTSSe | K |  | Spin coating +selenized | 180 nm | 52.2 | 7.37 | 7.04 |  |
|  | CZTSSe | K |  | Drop casting + selenized | 2.3 μm | 47.9 | 4.07 |  |  |
| 2 | CZTS | K | Particle | Solution+dip coating | / | 71.4 | 0.158 |  | [2] |
| 3 | CZTS | W | Particle | Drop-casting +500 °C for 30 min Ar annealing | 2.12 μm | 68.69 | 6.89 | 6.23 | [3] |
|  |  | K |  | Same | 2.2 μm | 65.72 | 4.89 |  |  |
| 4 | CZTSSe | K | 18–25 nm |  |  |  | 3.18 |  | [4] |
|  | CCoTSSe | K | 18–25 nm |  |  |  | 6.47 | 5.41 |  |
| Solvothermal | | | | | | | | | |
| 5 | CZTS | K | Porous thin film | solvothermal | 3 μm | 68.8 | 1.23 | 1.15 | [5] |
| 6 | CZTS | K | flower-like | Solvothermal+spin coating | 0.4 μm | 47 | 4.75 |  | [6] |
|  |  |  | 3% graphene |  |  | 60.4 | 6.93 |  |  |
|  |  |  | 2% graphene |  |  | 65.6 | 7.81 | 6.66 |  |
|  |  |  | 1% graphene |  |  | 52.4 | 5.53 |  |  |
| 7 | CZTS | K | Particle | solvothermal |  | 39.72 | 3.22 |  | [7] |
|  | CZTSSe | K |  | +selenized |  | 54.44 | 5.75 | 7.45 |  |
| 8 | CZTS | K | hierarchical | Solvothermal+spray painting  300 ^o^C air | 2 μm | 0.18 | 0.93 | 6.91 | [8] |
|  |  |  |  | 500 ^o^C N_2_ | 2 μm |  | 2.32 |  |  |
|  |  |  | 0.005M | 500 ^o^C S | 2 μm |  | 5.99 |  |  |
|  |  |  | 0.02M | 500 ^o^C S | 2 μm |  | 6.39 |  |  |
|  |  |  | 0.05M | 500 ^o^C S | 2 μm |  | 6.98 |  |  |
| 9 | CZTS | K |  | Solvothermal+blade-coating+500 ^o^C for 35 min in Ar | 4.8 μm | 73.06 | 7.63 |  | [9] |
|  | CZTSe | K |  |  | 5.1μm | 74.11 | 7.54 |  |  |
|  | CZTS/CZTSe | K |  |  | 5 μm | 72.96 | 8.83 | 8.49 |  |
| 10 | CFTS | K | 10 nm particle | Solvothermal  +drop casting+  sulfurized at 550 ^o^C for 30 min with S in Ar | 1.9 μm | 58 | 7.1 | 8.2 | [10] |
|  | CCTS | K | 12 nm particle |  | 2.2 μm | 60 | 7.4 |  |  |
| 11 | CZTS | K | particle | In-suit solvothermal | 580 nm | 63 | 5.65 | 4.96 | [11] |
|  |  |  |  |  | 350 nm | 37 | 3.15 |  |  |
| 12 | CZTS | K | Transparent Leaf-like Plate array | Electrodeposited+solvothermal | 350 nm | 57.2 | 7.09 | 6.01 | [12] |
|  |  |  |  | +mirror Reflect |  | 51.0 | 8.67 |  |  |
| 13 | CZTS | K | nanosheet networks | Electrodeposited+ solvothermal | 530 nm | 62.9 | 6.24 | 6.01 | [13] |
|  |  |  |  | +mirror Reflect | 530 nm | 62.1 | 7.12 |  |  |
| Solution method | | | | | | | | | |
| 14 | CZTS | K | Precursor Solution | Spin+300 ^o^C annealling |  |  |  |  | [14] |
|  |  |  |  | Selenization-  particle | 1.16 μm |  | 6.36 |  |  |
|  |  |  |  | bilayer | 1.18 μm |  | 6.86 |  |  |
|  |  |  |  | large grain | 1.20 μm |  | 7.43 | 6.58 |  |
| 15 | CZTS | K | Precursor Solution | Spray deposited at 350 ^o^C | ~ 1.0 μm | 48 | 3.7 |  | [15] |
|  |  |  |  | +H_2_S 500 ^o^C 1h | ~ 1.0 μm | 53 | 6.4 | 8.3 |  |
| 16 | CZTS | K | Sol-gel | Annealed in N_2_ at 550 ^o^C for 5 min | 200 nm | 59 | 5.63 | 5.44 | [16] |
| 17 | CZTS | K | Nanoflake  1.1 μm | successive ionic layer adsorption and reaction (SILAR) | 80 nm | 63 | 4.84 | 3.82 | [17] |
| Vacuum based methods | | | | | | | | | |
| 18 | CZTS | K | Nanoplate array | Pulsed laser deposition | 0.5 μm |  | 3.65 | 3.33 | [18] |
|  |  |  | Thin film | Pulsed laser deposition | 0.5 μm |  | 2.83 |  |  |
|  |  |  |  |  |  |  | 3.10 |  |  |
| 19 | CZTS | K | Thin film | Sputtered Cu-Zn-Sn sulfuring | 1.5 μm | 62.0 | 7.94 | 8.55 | [19] |

Table S2 Lattice parameters and energy for different arrangements of C1 to C4

|  | a(Å) | b(Å) | c(Å) | E(eV) |
| --- | --- | --- | --- | --- |
| C1 | 7.574 | 7.574 | 6.256 | 0.62 |
| C2 | 7.563 | 7.563 | 6.267 | 0.73 |
| C3 | 7.563 | 7.563 | 6.249 | 0 |
| C4 | 7.563 | 7.563 | 6.249 | 0 |

**References:**

Xin, X.; He, M.; Han, W.; Jung, J.; Lin, Z. *Angew. Chem. Int. Ed.* **2011**, *50* (49), 11739-11742.

Li , L.; Zhang, B. L.;Cao, M.; Sun, Y.; Jiang, J. C.; Hua, P. F.; Shen, Y.; Wang, L.J. *J. Alloys Compd.* **2013**, *551*, 24-29.

Kong, J.; Zhou, Z.- J.; Li, M.; Zhou, W.- H.; Yuan, S.- J.; Yao, R.- Y.; Zhao, Y.; Wu, S.- X. *Nanoscale Res. Lett.* **2013**, *8* (1), 464 1-5.

Özel, F.; Sarılmaz, A.; İstanbullu, B.; Aljabour, A.; Kuş, M.; Sönmezoğlu, S. *Sci. Rep.* **2016,** *6,* 29207.

Dai, P.; Zhang, G.; Chen, Y.; Jiang, H.; Feng, Z.; Lin Z.; Zhan, J.; *Chem. Commun.*, **2012**, *48*, 3006-3008.

L. Bai ; Ding, J.,N.; Yuan, N. Y.; Hu, H. W.; Li, Y.; Fang, X.; *Mater. Lett.*, **2013**, *112*, 219-222.

Shen, J.; Zhang, D.; Li, J.; Li, X.; Sun, Z.; Huang, S.; *Nano-Micro Lett.* **2013**, *5*(4), 281-288.

Xie, Y.; Zhang, C.; Yue, F.; Zhang, Y.; Shic, Y.; Ma, T. *RSC Adv.*,**2013**, *3*, 23264.

Zhu, L.; Qiang, Y. H.; Zhao, Y. L.; Gu, X. Q. Appl. Surf. Sci. **2014**, *292*, 55- 62.

Mokurala, K.; Mallick, S; Bhargava, P; *J. Power Sources* **2016**, *305*, 134e143.

Chen, S.; Xu, A.; Tao, J.; Tao, H.; Shen, Y.; Zhu, L.; Jiang, J.; Wang, T.; Pan, L. ACS Sustainable Chem. Eng. **2015**, *3*, 2652−2659.

Chen, S.- L.; Xu, A.- C.; Tao, J.; Tao, H.- J.; Shen, Y.- Z.; Zhu, L.- M.; Jiang, J.- J.; Wang, T.; Pan, L. *Green Chem.* **2016**, *18* (9), 2793-2801.

Chen, S.- L.; Tao, J.; Tao, H.- J.; Shen, Y.- Z.; Xu, A.- C.; Cao, F.- X.; Jiang, J.- J.; Wang, T.; Pan, L. *Dalton Trans.* **2016**, *45* (11), 4513-4517.

Chen, H.; Kou, D.; Chang, Z.; Zhou, W.; Zhou, Z.; Wu, S. *ACS Appl. Mater. Interfaces* **2014**, *6* (23), 20664-20669.

Swami, S. K.; Chaturvedi, N.; Kumar, A.; Chander, N.; Dutta, V.; Kumar, D. K.; Ivaturi, A.; Senthilarasu, S.; Upadhyaya, H. M. *Phys. Chem. Chem. Phys.* **2014**, *16*, 23993.

Tong, Z.; Su, Z.; Liu, F.; Jiang, L.; Lai, Y.; Li, J.; Liu, Y. *Mater. Lett.* **2014**, *121* (15), 241-243.

Mali, S. S.; Shim, C. S.; Hong, C. K. *Mater. Res. Bull.* **2014,** *59* (16), 249-253.

Wozny, S.; Wang, K.; Zhou, W. *J. Mater. Chem. A.* **2013**, *1* (48), 15517-15523.

Fan, M. S.; Chen, J. H.; Li, C. T.; Cheng, K. W.; Ho, K. C. *J. Mater. Chem. A.* **2014,** *3* (2), 562-569.
